# Supplementary figures and images for: Estimating the age of Heliconius butterflies from calibrated photographs
Source: PeerJ. 2017 Sep 27;5:e3821. doi: 10.7717/peerj.3821 (PMC5622606; doi:10.7717/peerj.3821)

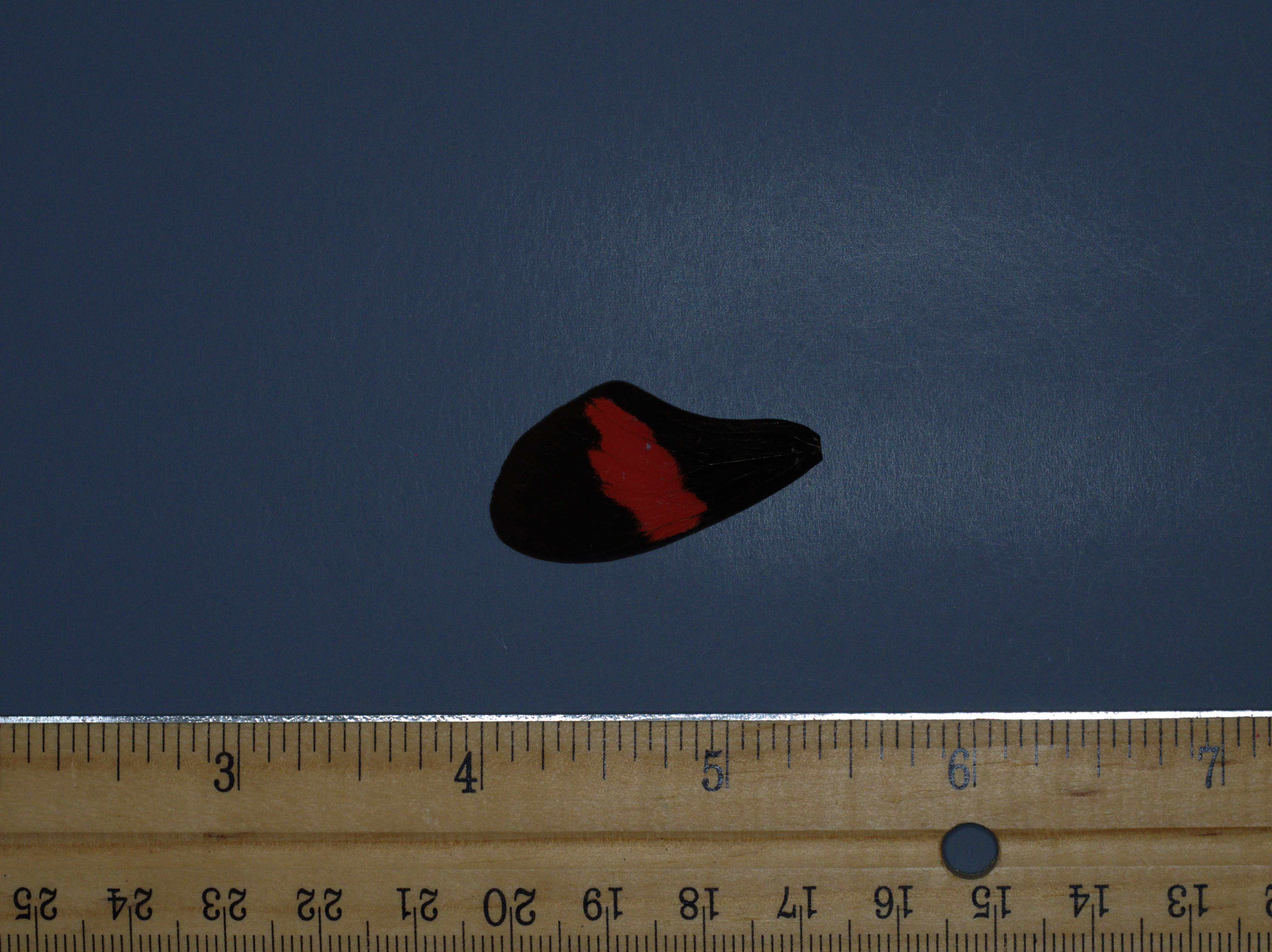

Supplement: Supplemental Information 1 — Shows dorsal forewing of Heliconius melpomene rosina from collection (#6216) used in this study. [file peerj-05-3821-s002.jpg]
